# Online Availability of Diamond Shruumz Before and After FDA Recall Initiation: Qualitative Assessment and Simulated Test Purchasing

**DOI:** 10.2196/64820

**Published:** 2025-06-30

**Authors:** Tim Mackey, Matthew Nali, Meng Zhen Larsen, Zhuoran Li, Jiawei Li, Joshua Yang

**Affiliations:** 1 Global Health Program, Department of Anthropology, University of California, San Diego, 9500 Gilman Drive, MC: 0505, La Jolla, CA, 92093, United States, 1 9514914161; 2 S-3 Research LLC, San Diego, CA, United States; 3 Global Health Policy and Data Institute, San Diego, CA, United States; 4 San Diego Supercomputer Center, La Jolla, CA, United States; 5 Department of Public Health, California State University, Fullerton, CA, United States

**Keywords:** online monitoring, psychedelics, social media, recall, qualitative assessment, psychoactive product, e-commerce, online surveillance, market, website

## Abstract

**Background:**

Reports of hospitalization associated with Diamond Shruumz–branded mushroom-containing products in October 2024 led to a manufacturer’s recall that restricted the sale, distribution, and accessibility of this new and emerging psychoactive product.

**Objective:**

This study seeks to assess the continuing online availability of a mushroom-containing edible product in a diverse e-commerce landscape, specifically aiming to identify and characterize its online availability before and after recall initiation.

**Methods:**

A retrospective online market surveillance of Diamond Shruumz products using structured and automated search queries was employed to identify online product marketing and availability. Online surveillance included the monitoring of multiple social media platforms, cannabis e-commerce websites, and search engine queries between June 22 and June 27, 2024, immediately preceding the manufacturer-initiated recall. Post-recall simulated purchases were then conducted on July 12, 2024, on platforms, websites and domains identified as continuing to actively market and sell the products through online product listings.

**Results:**

Prior to product recall, a total of 4117 product listings across 1600 (38.86%) social media posts and user-generated comments, 11 (0.27%) cannabis e-commerce websites, and 2509 (60.94%) hyperlinks from internet search queries were generated for further content analysis. Review of online sources revealed 49 social media posts, 8 e-commerce shops, and 67 domains that were identified as actively marketing and selling products prior to recall. Post-recall, we identified 45 (67.16%) remaining domains that continued to market the product from these different online sources. Simulated purchases revealed that 15 (33.33%) domains successfully transacted test purchases and 30 (66.66%) transactions failed because of account verification or payment failure.

**Conclusions:**

The Diamond Shruumz recall exemplifies the ongoing challenge of unknown consumer harm associated with new and emerging substances marketed and sold on the internet, which is especially concerning as these products appeal to younger audiences with a variety of edible flavored products. While a recall was initiated and products became unavailable, our study found that post-recall online vendors continued to market and sell the products. This indicates that there are ongoing challenges to effectuate recalls and online enforcement in a diverse e-commerce landscape that can rapidly bring new and novel psychoactive substances to the market.

## Introduction

As of October 31, 2014, the US Centers for Disease Control and Prevention (CDC) had reported 180 illnesses in 34 states, including 73 hospitalizations and 3 possible deaths, potentially associated with the consumption of the consumer edible brand, Diamond Shruumz [1,2].

Diamond Shruumz products, which include a diverse variety of flavored and infused cones, chocolate bars, and gummies, contain muscimol, a non–federally scheduled chemical derived from Amanita mushrooms (*Amania muscaria* [*A. muscaria*] is currently legal); however, batches of the product have been tested by the US Food and Drug Administration (FDA) and found to contain a designer drug synthetic analog (4-acetoxy-DMT [dimethyltryptamine], also known as *O*-acetylpsilocin or psilacetin) that can be equivalent to a Schedule 1 controlled substance under the Federal Analogue Act when sold for human consumption [2-4]. Other reports have characterized these products as being marketed as “nootropics” (substances taken to enhance cognitive function) and found that some contain unlabeled and undisclosed psilocybin and psilocin along with other compounds [2,5].

Diamond Shruumz products were sold across a variety of US retailers including tobacco shops, cannabis stores, and hemp-derived retailers and have been connected with a number of severe symptoms and adverse events reported to state poison control centers including seizures, loss of consciousness, nausea and vomiting, and hyper- and hypotension [6]. Though the manufacturer (Prophet Premium Blends, LLC) initiated a recall of these products on June 27, 2024, and agreed to cease production and distribution of the Diamond Shruumz product line, assessing if there was continued online availability and whether retailers were compliant with recall requirements has not been adequately studied [6,7].

Hence, in this rapid online market monitoring paper, we seek to examine the continued online availability of these products immediately after the product recall was initiated. To accomplish this, this study used a multistage protocol involving multiplatform online market surveillance, content analysis, and targeted simulated test purchases.

## Methods

### Overview

**Figure 1.**
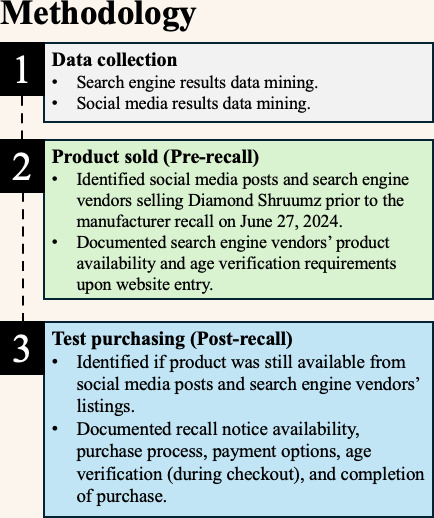
Summary of the study’s methodology, including the procedure for assessing online market availability, website monitoring, and simulated test purchasing after recall.

We conducted a qualitative retrospective online market surveillance study to assess whether Diamond Shruumz products continued to be marketed and made available for purchase online (see Figure 1 for a summary of the study’s methods). This included conducting structured and automated search queries on social media platforms (Instagram, Reddit, Telegram, Tumblr, and X [formerly known as Twitter]), cannabis product listings originating on e-commerce platforms (Dutchie, Leafly, and Weedmaps), and website hyperlinks generated from popular search engine results (Bing, DuckDuckGo, and Google). Automated searches were conducted from June 22 to June 27, 2024, immediately preceding the manufacturer-initiated recall (see Multimedia Appendix 1).

A qualitative review of social media posts, e-commerce product listings, and websites generated from search queries was conducted to identify active direct-to-consumer sale of any of the Diamond Shruumz products that were subject to recall. Where possible, we also attempted to assess if a seller was licensed by their respective state licensure board (eg, state cannabis control board). For websites actively advertising the sale of Diamond Shruumz products, analysis also included an assessment of any age verification processes and web forensics for internet sites using the Internet Corporation for Assigned Names and Numbers (ICANN) WHOIS look-up tool.

Finally, simulated test purchases in the period following the announcement of the recall were conducted to identify websites continuing to sell the products [8]. Simulated test purchases involved selecting a Diamond Shruumz product from an identified online seller that utilized traditional e-commerce tools (eg, hosted website, shopping cart, payment processing), placing it in an e-commerce shopping cart, proceeding through the purchase process, and simulating the purchase transaction using a credit card number generator that has been used in previous studies for e-commerce testing and test buy purposes [9-11]. Completion or denial of the transaction was documented. Qualitative data analysis and simulated purchases were performed on July 12, 2024, 2 weeks after the manufacturer initiated the recall notice.

### Ethical Considerations

This study utilized publicly available data and did not involve human participants or any personally identifiable information from individuals or online sellers; it therefore did not require ethics approval. All information collected from this study was from the public domain and the study did not involve any interaction with users. Any identifiable user information was aggregated and removed from the study’s results.

## Results

### Overview

Market surveillance detected a total of 4117 Diamond Shruumz product listings available across all parts of the internet monitored. Product listings potentially selling Diamond Shruumz products were found in 1600 (38.86%) social media posts and comments, 11 (0.27%) cannabis e-commerce websites, and 2509 (60.94%) hyperlinks from internet search queries from the period immediately preceding the initiation of the recall (see Table 1 for a summary of the platform breakdown results, Table 2 for examples of active selling posts detected after the initiation of recall, and Table S1 in Multimedia Appendix 1 for the deidentified results of simulated test purchases).

**Table 1.** Summary of social media queries, cannabis e-commerce platforms, and internet search queries for Diamond Shruumz products.

Platforms	Total aggregate, n	Advertised product listings (before FDA^a^ recall), n (%)	Simulated purchases (after FDA recall), n (%)
Social media queries			
Instagram posts	1103	33 (67.35)	0 (0)^b^
Reddit posts	20	0 (0)	0 (0)
Telegram posts	2	2 (4.08)	0 (0)^b^
Tumblr posts	16	0 (0)	0 (0)
X posts	399	14 (28.57)	1 (100)
Instagram user-generated comments	52	0 (0)	0 (0)
Reddit user-generated comments	8	0 (0)	0 (0)
Total social media queries	1600	49 (100)	1 (100)
Cannabis e-commerce websites			
Dutchie shops	1	1 (12.50)	1 (50)
Leafly shops	5	5 (62.50)	0 (0)
Weedmaps shops	2	2 (25)	1 (50)
Total e-commerce shops	8	8 (100)	2 (100)
Internet search queries			
Unique domains	237	67 (28.27)	15 (33.33)

a FDA: US Food and Drug Administration.

b Original signal product listing unavailable.

**Table 2.** Screenshot examples of simulated purchases conducted on social media, e-commerce listings, and internet search queries post manufacturer-initiated recall.

	Platform	Example
Social media posts	X	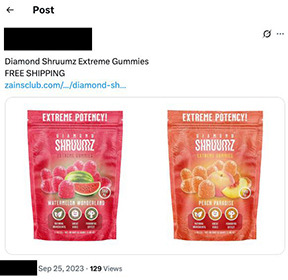
Cannabis e-commerce product listing	Dutchie	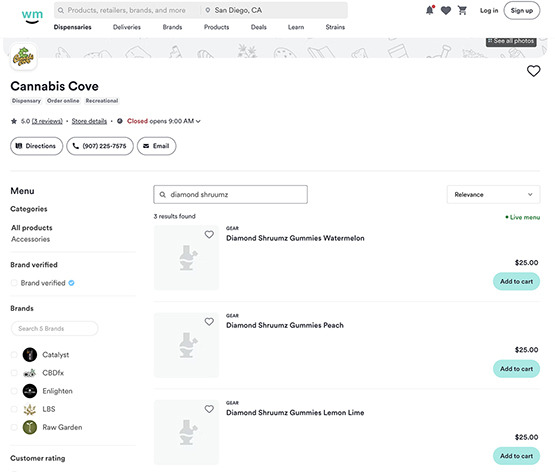
Internet search queries domain	Prime Chocolate Store	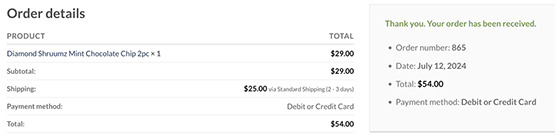

### Social Media Queries

Automated search queries on select social media platforms (Instagram, Reddit, Telegram, Tumblr, and X) identified 1600 Diamond Shruumz product listings, where 96.25% (n=1540) originated from user-generated posts and 3.75% (n=60) were from user-generated comments across only 2 platforms (Instagram and Reddit). Direct-to-consumer advertisements with links or contact information that users could use to directly purchase the products made up 3.06% (n=49) of posts, of which 67.35% (n=33) originated from Instagram, 28.57% (n=14) from X, and 4.08% (n=2) from Telegram. Simulated purchases were conducted on the social media posts, where only 1 social media advertisement, detected on X, was still actively selling Diamond Shruumz products after recall initiation.

### Cannabis e-Commerce Product Listings

Structured search queries on cannabis e-commerce product listing websites (Dutchie, Leafly, and Weedmaps) detected a total of 8 product listings (belonging to 4 unique cannabis vendors) that advertised Diamond Shruumz products via these platforms. Cannabis e-commerce platform vendor breakdown of these listings included Dutchie (n=1, 12.5%), Leafly (n=5, 62.5%), and Weedmaps (n=2, 25%). All vendor listings on Dutchie and 1 vendor listing from Weedmaps were linked to the same licensed cannabis retailer in Alaska. The remaining Weedmaps listing was linked to an expired licensed cannabis retailer in Oklahoma. Leafly store listings were linked to 2 shops: one in Hawaii that was verified to have an active cannabis license and one in Wisconsin that could not be verified due to lack of access to publicly available state licensure data. Post-recall simulated purchases identified the Alaska storefront as still actively selling Diamond Shruumz products.

### Structured Internet Search Queries

For internet search queries, a total of 2509 hyperlinks, comprising 237 unique domain names, were collected; 67 (28.27%) domains were identified as actively selling Diamond Shruumz products prior to recall. From the sellers with sufficient information to cross-reference available state cannabis licensure data, none were linked to a valid retail cannabis state license. All websites that were actively selling Diamond Shruumz went through web forensics analysis. The majority (n=41, 61.19%) included registrant information located in the United States (including Arizona, Pennsylvania, and California as the top 3 states), followed by Canada and Iceland, with 2 websites also using domain privacy services to hide their information. Slightly over half (n=37, 55.22%) of the reviewed sites had a form of age verification, with those that did requiring a single, nonrestricted click confirming that a user was over the age of 18 years (or 21 years, based on applicable laws). Post recall, 67.16% (n=45) of re-reviewed websites continued to advertise the sale of Diamond Shruumz product. One-third (n=15, 33.33%) of those websites continued to offer the direct sale of a product (ie, where transaction and simulated purchase was successful) while two-thirds (n=30, 66.66%) of transactions failed to process (ie, payment not accepted, used robust account verification).

## Discussion

### Principal Findings

Results from this study indicate that the time period immediately preceding the manufacturer-initiated recall (a period when the US FDA, America’s Poison Centers, and CDC identified initial concerns about illnesses associated with consuming these products and actively provided updates to the public) had a relatively high number of product advertisements available across multiple parts of the internet that likely led to high consumer exposure to these products. Following the recall notification, the number of online access points appeared to decrease; however, we nevertheless identified at least 47 active sellers across social media, cannabis e-commerce, and website platforms.

Similar to prior studies examining online availability of psilocybin [12], there were a high number of continued access points that originated from search engine results that linked to websites selling direct-to-consumer products, two-thirds of which were found to be actively advertising even during the post-recall period. For approximately one-third of those websites, we were able to successfully complete a simulated test purchase, and some vendors actively reached out through email seeking to complete a cancelled order we initiated through this process.

The Diamond Shruumz product recall notification exemplifies some of the ongoing challenges of consumer harms associated with new and emerging substances that become readily available on the internet; in this case, novel edible mushroom-containing nootropics. This incident also has a similar fact pattern to the 2019 outbreak of e-cigarette or vaping product use–associated lung injury, which ended with a total of 2807 hospitalized cases (68 of which were deaths) attributed to vitamin E acetate in tetrahydrocannabinol (THC)-containing nicotine products [12]. In the case of Diamond Shruumz, interest in psychedelics and psilocybin therapy is growing [13-15], though psilocybin remains illegal, and products mimicking psychedelic effects or possibly containing microdoses of psychoactive properties appear to be available via direct-to-consumer sales, despite unknown potential health risks.

To capitalize on the growing interest but also the slow path of potential legalization of psychedelics, manufacturers may be experimenting with undeclared and unlabeled synthetic analogues for human consumption, such as 4-acetoxy-DMT [3,16]. Further, the FDA has warned food manufacturers that *A. muscaria*’s extracts and certain constituents, as found in Diamond Shruumz samples tested by the FDA, do not meet Generally Recognized as Safe (GRAS) standards, are not authorized for use as ingredients in foods, and may be harmful [2,17]. Alarmingly, Diamond Shruumz products also included appealing marketing of diverse edible forms (eg, gummies, bars, cones) and flavors (eg, chocolate, birthday cake, various fruit flavors) found with other increasingly publicly acceptable substances, such as cannabis and nicotine products, which may be attractive to children and young adults [18].

Continued availability of these products, which we observed in the early stages of initiation of this recall, highlights the challenges of effectuating recalls in a diverse e-commerce landscape for a novel product that has legal ambiguity (ie, *A. muscaria* is legal and 4-acetoxy-DMT is unscheduled, but DMT and psilocybin are scheduled and prohibited by the US Drug Enforcement Administration). Reflecting these challenges, the FDA announced that it was working with the National Association of Convenience Stores and the National Smoke Shop Association to raise consumer awareness about the recall and warn consumers about purchasing or consuming Diamond Shruumz [2,19-21].

### Limitations

Limitations of this study include the relatively short time period after recall initiation to conduct market surveillance and simulated test purchases, legal and ethical limitations that restricted our ability to conduct real-world test buys of actual products that necessitated simulated purchasing instead, and lack of complete licensure data to assess all vendor status. Future studies should address these limitations by assessing the practicality of conducting legally authorized test purchases that involve package and analytical chemical testing analysis as has been done in prior studies for other types of medications [22,23].

### Conclusions

This paper aimed to conduct a rapid market surveillance study to identify ongoing consumer and public health risks associated with the recall of Diamond Shruumz products that were sold throughout the United States via a variety of online channels. The public health urgency of these findings is emphasized by other observations we had, such as our detection of 12 social media user-generated posts discussing purported adverse events, including a self-reported hallucination and severe adverse health experience after consuming 2 Diamond Shruumz chocolate bars by one online user (see Figure S1 in Multimedia Appendix 1). This emphasizes the need for continued surveillance in a post-recall period, particularly in the context of the concerning safety profile of *A. muscaria* and its toxic compounds, such as muscimol, which was detected in Diamond Shruumz products and has the potential for serious adverse events [15]. Further, there is a need for novel and persistent online marketplace monitoring approaches coupled with proactive postmarket surveillance (eg, pharmacovigilance, nutravigilance, and cannabis product surveillance), particularly for consumer groups who may purchase online and not report their adverse event experiences to poison control centers or do not end up hospitalized [19-21,24]. Customized approaches using artificial intelligence and multiplatform analysis will be key to these efforts as new products emerge in virtual spaces.

## Supplementary material

10.2196/64820 Multimedia Appendix 1 Additional information regarding study methods, simulated purchases of Diamond Shruumz products, and a Reddit user’s severe adverse health experience.

## Abbreviations

*A. muscaria*: Amania muscaria
CDC: Centers for Disease Control and Prevention
DMT: dimethyltryptamine
FDA: Food and Drug Administration
GRAS: Generally Recognized as Safe
ICANN: Internet Corporation for Assigned Names and Numbers
THC: tetrahydrocannabinol

## References

[1] Severe illness potentially associated with consuming Diamond ShruumzTM brand chocolate bars, cones, and gummies. US Centers for Disease Control and Prevention.

[2] Investigation of illnesses: Diamond Shruumz-brand chocolate bars, cones, & gummies (June 2024). US Food and Drug Administration.

[3] 21 US Code § 813 - treatment of controlled substance analogues. Cornell Law School.

[4] (2022). *N,N*-Dimethyltryptamine (DMT). https://deadiversion.usdoj.gov/drug_chem_info/dmt.pdf.

[5] Michienzi A, Hamlin J, Farah R, Bazydlo L (2024). Notes from the field: Schedule I substances identified in nootropic gummies containing Amanita muscaria or other mushrooms - Charlottesville, Virginia, 2023-2024. MMWR Morb Mortal Wkly Rep.

[6] Prophet premium blends recalls Diamond Shruumz products because of possible health risk. US Food and Drug Administration.

[7] Diamond Shruumz.

[8] Whois lookup. DomainTools.

[9] Nali MC, Purushothaman V, Li Z, Cuomo R, Mackey TK (2023). Assessing the impact of the Massachusetts temporary flavor ban on licensed tobacco retailers. Tob Use Insights.

[10] Nali MC, Purushothaman V, Xu Q, Cuomo RE, Mackey TK (2021). Characterizing and assessing compliance of online vendors to the state of Massachusetts ENDS product sales ban. Tob Induc Dis.

[11] Dummy / fake credit card generator. SEO Expert Melbourne Saijo George.

[12] Outbreak of lung injury associated with the use of e-cigarette, or vaping, products. US Centers for Disease Control and Prevention.

[13] Yerubandi A, Thomas JE, Bhuiya N, Harrington C, Villa Zapata L, Caballero J (2024). Acute adverse effects of therapeutic doses of psilocybin: a systematic review and meta-analysis. JAMA Netw Open.

[14] Kupferschmidt K In a setback for psychedelic therapy, FDA advisers vote against medical use of ecstasy. Science.

[15] Leas EC, Satybaldiyeva N, Kepner W (2024). Need for a public health response to the unregulated sales of Amanita muscaria mushrooms. Am J Prev Med.

[16] Wainer D (2024). Psychedelics are going mainstream: investing in them hasn’t. The Wall Street Journal.

[17] FDA alerts industry and consumers about the use of Amanita muscaria or its constituents in food. US Food and Drug Administration.

[18] Nali MC, Yang JS, Li Z, Larsen MZ, Mackey TK (2024). Cannabis-derived product types, flavors, and compound types from an e-commerce website. JAMA Netw Open.

[19] Nali MC, Purushothaman V, Li J, Mackey TK (2022). Characterizing California licensure status and tobacco user experience with adverse events using Yelp data. Prev Med Rep.

[20] Yang JS, Lim P, Ojeda K, Cuomo RE, Purushothaman V, Mackey T (2022). Inductive characterization of ENDS-associated adverse events among California young adults. AJPM Focus.

[21] Purushothaman V, McMann TJ, Li Z, Cuomo RE, Mackey TK (2022). Content and trend analysis of user-generated nicotine sickness tweets: a retrospective infoveillance study. Tob Induc Dis.

[22] Ashraf AR, Mackey TK, Vida RG (2024). Multifactor quality and safety analysis of semaglutide products sold by online sellers without a prescription: market surveillance, content analysis, and product purchase evaluation study. J Med Internet Res.

[23] Mackey TK, Jarmusch AK, Xu Q (2022). Multifactor quality and safety analysis of antimicrobial drugs sold by online pharmacies that do not require a prescription: multiphase observational, content analysis, and product evaluation study. JMIR Public Health Surveill.

[24] Schmitz SM, Lopez HL, MacKay D (2014). Nutravigilance: principles and practices to enhance adverse event reporting in the dietary supplement and natural products industry. Int J Food Sci Nutr.

