# Supplementary material for: Online Availability of Diamond Shruumz Before and After FDA Recall Initiation: Qualitative Assessment and Simulated Test Purchasing
Source: J Med Internet Res. 2025 Jun 30;27:e64820. doi: 10.2196/64820 (PMC12232493; doi:10.2196/64820)
Supplement: Multimedia Appendix 1 [file jmir-v27-e64820-s001.docx]

**Supplemental Online Content**

This supplemental material has been provided by the authors to give readers additional information about their work.

eMethods.

**Methods:**

**Data Collection:**

Market surveillance data collection used structured and automatic queries from popular search engine browsers (Bing, DuckDuckGo and Bing) and social media platforms (Instagram, Reddit, Tumblr, Twitter, and YouTube) to catalog websites and social media content using commercially available proxy services and customized data mining approaches to collect publicly available data from the Internet developed with Python (3.8) and Selenium (4.15.1). These data mining tools operated on five keywords mentioned in the FDA investigation on the Diamond Shruumz brand, emulating typical user behavior by inputting these keywords into search fields on various platforms and search engines. After completion, the scrapers returned relevant data of specific data features for each of the platforms searched including: “social media links”, “domain links”, “social media platform IDs”, “social media post and comment text description”, “website text description”, “username”, and “timestamps”. Structured search engine queries captured data from the top 45 pages displayed by the search engine multiple times.

**Diamond Shruumz Keywords:**

“Diamond Shruumz”, “Shruumz”, “Shruumz chocolate bars”, “Shruumz Gummies”, and “Shruumz Cones”

**Table S1. Simulated purchases of Diamond Shruumz products on unique online vendor characteristics offered online (site domain names de-identified).**

| **Domain (location^a^)** | **Registrar** | **Seller Type** | **Recall Notice** | **Age Verification (Entering Website)** | **Diamond Shruumz Availability (Simulated purchase)** | **Product price range^b^** | **Payment options** | **Age Verification (During Simulated purchase)** | **Final status of simulated purchase** |
| --- | --- | --- | --- | --- | --- | --- | --- | --- | --- |
| **Website 1 (United States)** | FastDomain Inc. | Wholesaler | No | Yes (Mono 21+) | Yes (Bulk) | N/a | Visa, Mastercard, AMEX, Discover, Google Pay, Apple Pay, Amazon | Yes | Account needs verification |
| **Website 2 (United States)** | GoDaddy.com, LLC | Store | No | Yes (Binary 18+) | Yes (Individual) | $25 | Visa, Mastercard, AMEX, Bank Transfer | No | Payment not accepted |
| **Website 3 (United States)** | TLD Registrar Solutions Ltd. | Store | No | None | Yes (Individual) | $23 | Visa, Mastercard, PayPal, Cash | No | Completed |
| **Website 4 (Canada)** | TUCOWS, INC. Tucows Domains Inc. | Store | No | Yes (Binary 21+) | Yes (Individual) | $25.99-$31.19 | Visa, Mastercard, AMEX, Discover | No | Payment not accepted |
| **Website 5 (United States)** | Hostinger Operations, UAB | Store | No | None | Yes (Both) | $25-$229 | Bank Transfer, Google Pay | No | Completed |
| **Website 6 (United States)** | Namesilo, LLC | Wholesaler | No | None | Yes (Both) | $29-$124 | Visa, Mastercard, Apple Pay, PayPal, Venmo, Bitcoin | No | Completed |
| **Website 7 (Malaysia)** | WEBCC Web Commerce Communications Limited dba WebNic.cc | Wholesaler | No | None | Yes (Both) | $29-$259.99 | Bank Transfer, CashApp, Bitcoin | No | Completed |
| **Website 8 (United States)** | Squarespace Domains II LLC | Wholesaler | No | Yes (Binary 21+) | Yes (Bulk) | $27.99-$29.99 | Visa, Mastercard, AMEX, Discover | No | Payment not accepted |
| **Website 9 (United States)** | GoDaddy.com, LLC | Store | No | Yes (Age Insert 18+) | Yes (Individual) | $29 | Visa, Mastercard, AMEX, Discover | No | Payment not accepted |
| **Website 10 (United States)** | IONOS SE | Store | No | Yes (Age Insert 21+) | Yes (Individual) | $25-$40 | Cash, Online Payment through email | No | Completed |
| **Website 11 (United States)** | NameSilo, LLC | Wholesaler | No | Yes (Binary 18+) | Yes (Both) | $25-$760 | Bank Account, Cash, Google Pay, Apple Pay, Bitcoin | No | Completed |
| **Website 12 (Canada)** | TUCOWS, INC. Tucows Domains Inc. | Store | No | None | Yes (Individual) | $30 | Visa, Mastercard, AMEX, Discover | No | Payment not accepted |
| **Website 13 (Cameroon)** | TUCOWS, INC. Tucows Domains Inc. | Store | No | None | Yes (Individual) | $50 | Visa, Mastercard, Apple Pay, Cash, Bitcoin | No | Completed |
| **Website 14 (United States)** | Hostinger Operations, UAB | Store | No | None | Yes (Individual) | $35 | Bank Transfer, CashApp, Bitcoin | No | Completed |
| **Website 15 (Canada)** | TUCOWS, INC. Tucows Domains Inc. | Store | No | Yes (Binary 21+) | Yes (Individual) | $20.48-$64.99 | Visa, Mastercard, AMEX, Discover, Sezzle | No | Payment not accepted |
| **Website 16 (Iceland)** | NAMECHEAP INC NameCheap, Inc. | Store | No | None | Yes (Individual) | $30 | N/a | No | Order Error |
| **Website 17 (United States)** | PDR Ltd. d/b/a PublicDomainRegistry.com | Wholesaler | No | Yes (Binary 21+) | Yes (Bulk) | N/a | Bank Account, Cash | No | Order Error |
| **Website 18 (United States)** | GoDaddy.com, LLC | Wholesaler | No | None | Yes (Bulk) | N/a | N/a | Yes | Account needs verification |
| **Website 19 (United States)** | Name.com, Inc. | Store | No | Yes (Binary 21+) | Yes (Individual) | $25.99-$29.99 | Visa, Mastercard, AMEX, Discover | No | Payment not accepted |
| **Website 20 (United States)** | GoDaddy.com, LLC | Wholesaler | No | Yes (Binary 21+) | Yes (Bulk) | $140 | Visa, Mastercard, AMEX, PayPal | Yes | Account needs verification |
| **Website 21 (United States)** | GoDaddy.com, LLC | Store | No | None | Yes (Individual) | $38 | Visa, Mastercard, AMEX, PayPal | No | Individual seller from e-commerce platform |
| **Website 22 (United States)** | NameSilo, LLC | Store | No | None | Yes (Both) | $25-$29 | Debit/Credit (Unspecified) | No | Completed |
| **Website 23 (United States)** | GoDaddy.com, LLC | Wholesaler | No | Yes (Binary 21+) | Yes (Individual) | $12.50-$16.99 | Visa, Mastercard, AMEX, Discover | No | Need EIN/Tax ID to Create Account |
| **Website 24 (United States)** | Squarespace Domains II LLC | Wholesaler | No | None | Yes (Individual) | N/a | Visa, MasterCard, AMEX, Money Order, Check | No | Need EIN/Tax ID to Create Account |
| **Website 25 (Iceland)** | NAMECHEAP INC NameCheap, Inc. | Store | No | None | Yes (Both) | $25-$229 | Cashapp, Zelle | No | Completed |
| **Website 26 (United States)** | DREAMHOST DreamHost, LLC | Store | No | None | Yes (Both) | $25-$229 | Cashapp, Zelle, E-Transfer, Bitcoin | No | Completed |
| **Website 27 (United States)** | GoDaddy.com, LLC | Wholesaler | No | Yes (Binary 21+) | Yes (Both) | N/a | N/a | No | Need EIN/Tax ID to Create Account |
| **Website 28 (United States)** | TUCOWS, INC. Tucows Domains Inc. | Store | No | Yes (Binary 21+) | Yes (Individual) | $29.99 | Visa, Mastercard, AMEX, Discover | No | Payment not accepted |
| **Website 29 (United States)** | IONOS SE | Store | No | None | Yes (Individual) | $29.99-$34.99 | Visa, Mastercard, AMEX, Discover | No | Completed |
| **Website 30 (Redacted)** | GoDaddy.com, LLC | Store | No | None | Yes (Individual) | $29.99 | Visa, Mastercard, AMEX, Discover | No | Payment not accepted |
| **Website 31 (United States)** | Key-Systems GmbH | Store | No | None | Yes (Bulk) | $120-$600 | Cashapp, Zelle, Apple Pay, Google Pay, Chime, Venmo, Paypal | No | Completed |
| **Website 32 (Iceland)** | NAMECHEAP INC NameCheap, Inc. | Store | No | None | Yes (Both) | $25-$229 | Cashapp, Zelle, PayPal, Bitcoin | No | Completed |
| **Website 33 (United States)** | ENOM, INC. eNom, LLC | Store | No | None | Yes (Individual) | $19.99-$23.99 | Visa, Mastercard, AMEX, Discover, Google Pay | No | Payment not accepted |
| **Website 34 (United States)** | IONOS SE | Store | No | Yes (Binary 21+) | Yes (Individual) | $29.99 | Visa, Mastercard, AMEX, Discover | No | Payment not accepted |
| **Website 35 (Canada)** | TUCOWS, INC. Tucows Domains Inc. | Store | No | Yes (Binary 21+) | Yes (Individual) | $25.99-$31.19 | N/a | Yes | Submit Photo ID |
| **Website 36 (Canada)** | TUCOWS, INC. Tucows Domains Inc. | Wholesaler | No | None | Yes (Individual) | $20-$50 | N/a | Yes | Submit Photo ID |
| **Website 37 (United States)** | GoDaddy.com, LLC | Store | No | Yes (Binary 18+) | Yes (Individual) | $37.99 | Visa, Mastercard, AMEX, JCB | No | Payment not accepted |
| **Website 38 (United States)** | GoDaddy.com, LLC | Store | No | Yes (Binary 21+) | Yes (Individual) | $19.99-$24.99 | Visa, Mastercard, AMEX, JCB, eCheck, Bitcoin | No | Payment not accepted |
| **Website 39 (United States)** | GoDaddy.com, LLC | Wholesaler | No | Yes (Binary 21+) | Yes (Individual) | N/a | N/a | No | Need EIN/Tax ID to Create Account |
| **Website 40 (United States)** | GoDaddy.com, LLC | Store | No | Yes (Binary 21+) | Yes (Individual) | $24.99-$25.99 | Visa, Mastercard, AMEX, Discover | No | Payment not accepted |
| **Website 41 (Iceland)** | NAMECHEAP INC NameCheap, Inc. | Store | No | None | Yes (Both) | $19.95-$229.99 | Visa, Mastercard, AMEX, Discover | No | Completed |
| **Website 42 (United States)** | GoDaddy.com, LLC | Wholesaler | No | Yes (Binary 21+) | Yes (Bulk) | N/a | Visa, Mastercard, AMEX, Discover | No | Need EIN/Tax ID to Create Account |
| **Website 43 (United States)** | GoDaddy.com, LLC | Store | No | Yes (Binary 21+) | Yes (Individual) | $15.99-$19.99 | Visa, Mastercard | No | Payment not accepted |
| **Website 44 (United States)** | GoDaddy.com, LLC | Store | No | Yes (Binary Legal Age) | Yes (Bulk) | $119.99-$225.99 | Visa, Mastercard, AMEX, Discover | Yes | Submit Photo ID |
| **Website 45 (United States)** | GoDaddy.com, LLC | Store | No | Yes (Binary 21+) | Yes (Individual) | $24.99 | Visa, Mastercard, AMEX, JCB | No | Payment not accepted |

a: Purported location listed on ICANN WHOIS; b: Price range of one Diamond Shruumz product offered for sale.
Abbreviations: N/A: Not Available

**Figure S1: Reddit Users reporting self-reported adverse event after consuming Diamond Shruumz**

**
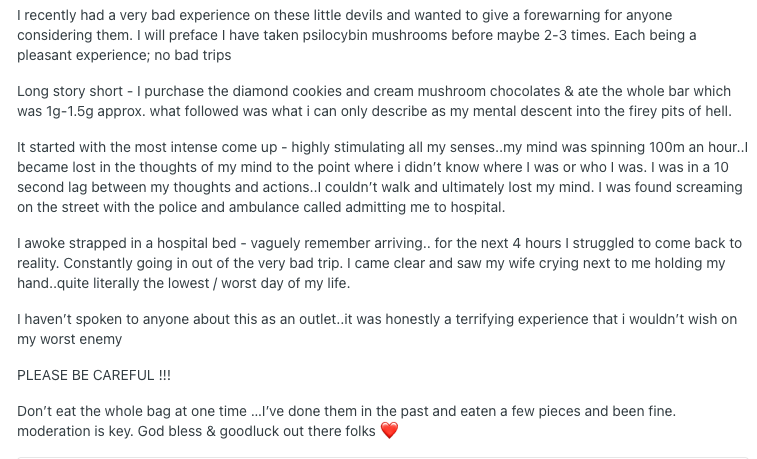
**
